# The Many Dimensions of Diet Breadth: Phytochemical, Genetic, Behavioral, and Physiological Perspectives on the Interaction between a Native Herbivore and an Exotic Host

**DOI:** 10.1371/journal.pone.0147971

**Published:** 2016-02-02

**Authors:** Joshua G. Harrison, Zachariah Gompert, James A. Fordyce, C. Alex Buerkle, Rachel Grinstead, Joshua P. Jahner, Scott Mikel, Christopher C. Nice, Aldrin Santamaria, Matthew L. Forister

**Affiliations:** 1 Program in Ecology, Evolution, and Conservation Biology, Department of Biology, University of Nevada, Reno, NV, 89557, United States of America; 2 Department of Biology, University of Nevada, Reno, NV, 89557, United States of America; 3 Department of Biology, Utah State University, Logan, UT, 84322, United States of America; 4 Department of Ecology and Evolutionary Biology, University of Tennessee, Knoxville, TN, 37996, United States of America; 5 Department of Botany, University of Wyoming, Laramie, WY, 82071, United States of America; 6 Department of Biology, Texas State University, San Marcos, TX, 78666, United States of America; University of Arkansas, UNITED STATES

## Abstract

From the perspective of an herbivorous insect, conspecific host plants are not identical, and intraspecific variation in host nutritional quality or defensive capacity might mediate spatially variable outcomes in plant-insect interactions. Here we explore this possibility in the context of an ongoing host breadth expansion of a native butterfly (the Melissa blue, *Lycaeides melissa*) onto an exotic host plant (alfalfa, *Medicago sativa*). We examine variation among seven alfalfa populations that differed in terms of colonization by *L*. *melissa*; specifically, we examined variation in phytochemistry, foliar protein, and plant population genetic structure, as well as responses of caterpillars and adult butterflies to foliage from the same populations. Regional patterns of alfalfa colonization by *L*. *melissa* were well predicted by phytochemical variation, and colonized patches of alfalfa showed a similar level of inter-individual phytochemical diversity. However, phytochemical variation was a poor predictor of larval performance, despite the fact that survival and weight gain differed dramatically among caterpillars reared on plants from different alfalfa populations. Moreover, we observed a mismatch between alfalfa supporting the best larval performance and alfalfa favored by ovipositing females. Thus, the axes of plant variation that mediate interactions with *L*. *melissa* depend upon herbivore life history stage, which raises important issues for our understanding of adaptation to novel resources by an organism with a complex life history.

## Introduction

Dietary niche breadth, or the number and type of resources consumed by an organism, drives numerous ecological and evolutionary processes, from mediating the coexistence of competitors [1, 2] to predicting geographical range size [3]. Herbivorous insects are useful in the study of dietary niche breadth because their lives are often so closely tied to their host plants that any change in diet can affect multiple aspects of the insect’s life history [4, 5, 6]. Moreover, associations between insects and their host plants are labile across time and space, and we would like to understand the causes and consequences of changes in host breadth [7, 8, 9, 10, 11, 12].

Dietary niche breadth of herbivorous insects is necessarily dependent on host plant variation, the importance of which has typically been explored in a comparative fashion across plant taxa. This comparative work has laid the foundations of plant defense theory and has generated many hypotheses for how and why insects use a particular plant species (e.g. physiological efficiency [5, 13], neural limitation [14], and enemy-free space [15]). From the perspective of an herbivorous insect, however, conspecific host individuals are not identical, and intraspecific variation in plant phytochemistry or nutrition might, in some cases, influence insect behavior and fitness as much as interspecific variation (reviewed in [16, 17, 18, 19]). The utility of studying intraspecific variation has been made clear by the rise of community genetic studies, which have demonstrated the importance of plant genetic variation for arthropod community assembly [20, 21, 22, 23]. Variation in phytochemistry or nutritional quality among conspecific hosts has also been linked to arthropod community assembly [24, 25, 26], but studies linking intraspecific host variation to arthropod behavior or performance are still uncommon compared to studies that focus on interspecific differences among plant taxa [27, 28, 29]. Here we explore the consequences of intraspecific host variation in the context of an ongoing expansion of dietary niche breadth using the butterfly *Lycaeides melissa* and its introduced host, alfalfa (*Medicago sativa*).

*L*. *melissa* is a widespread butterfly in western North America, where it feeds on Fabaceous plants (the pea family). Within the last 200 years, *L*. *melissa* has expanded its host range to include the non-native legume alfalfa [30]. *L*. *melissa* is most often found in association with alfalfa in disturbed areas, such as along roadsides and fallow fields. Alfalfa is a poor resource for caterpillars, leading to the development of adults that are up to 70% smaller than those reared on native hosts [31]. The poor quality of alfalfa can be at least partially mitigated by the presence of mutualistic ants [32], which deter enemies, and by flowering phenology, as flowers are a better larval food source than leaves [31]. The use of alfalfa by *L*. *melissa* in the wild is heterogeneous, with a majority of alfalfa populations in a region being unoccupied, at least in the arid Great Basin of western North America. Local adaptation by *L*. *melissa* to alfalfa has been detected [33], and differences among alfalfa-associated populations of *L*. *melissa* have been observed in larval performance [34] and adult preference [35]. However, in contrast to these studies demonstrating inter-population variation in butterflies, variation among alfalfa populations, and its importance to butterflies, has not been studied.

Cultivars of *M*. *sativa* are typically genetically diverse, because of the numerous parents utilized during the cultivar breeding process, and because desirable plant traits are negatively affected by inbreeding [36] This genetic variation underlies differences in plant traits among cultivars [37] including foliar protein [38], phytochemistry (e.g. saponin concentrations [39,40]), and pest resistance [41, 42, 43, 44]. Given this phenotypic and genetic variation among cultivated alfalfa, it is reasonable to expect variation in phytochemistry and foliar protein among patches of wild alfalfa utilized by *L*. *melissa*.

**Fig 1:**
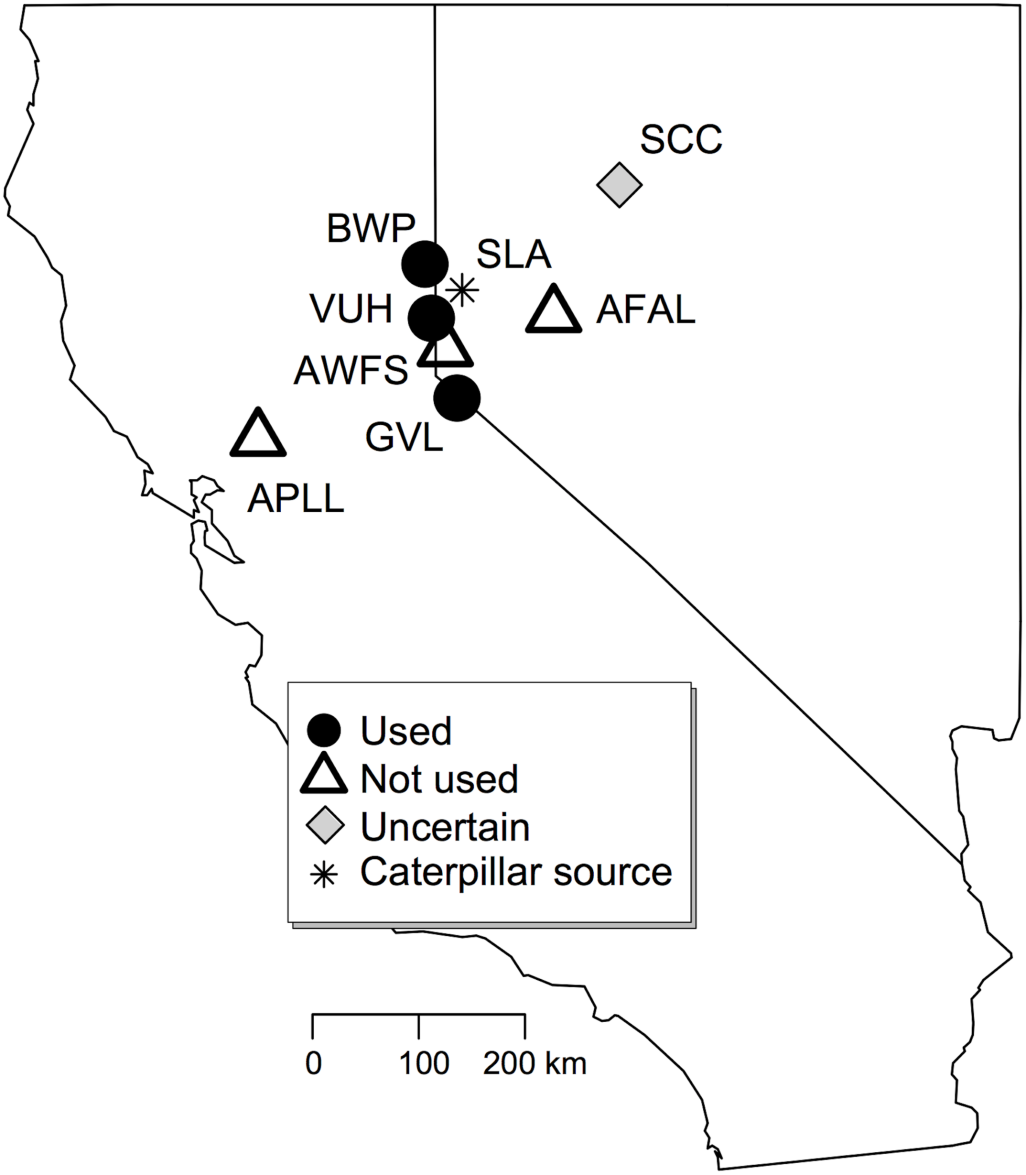
Map of locations from which alfalfa was collected for experiments (states pictured are California and Nevada in the western United States). Status (used or not used) indicates whether alfalfa locations support butterflies in the field. Unused locations are prefixed by the letter “A.” The uncertain status (for SCC) indicates a location where butterflies have been observed in the presence of alfalfa and a native host, but oviposition on alfalfa has not been confirmed. The caterpillar source (SLA) indicated by the asterisk is the location of origin for caterpillars used in performance experiments. Females for preference tests came from both SLA and VUH. For descriptions of each study location see main text.

Here we characterize variation in herbivore performance and behavior in response to different host populations with the goal of understanding how performance and behavior might be predicted by host plant traits. To do so, we examined seven alfalfa populations with a known history of *L*. *melissa* presence or absence (Fig 1). Six of these alfalfa populations are located within the geographic range of *L*. *melissa*, and the seventh is beyond the western range limit, in the Central Valley of California. We chose alfalfa populations differing in terms of *L*. *melissa* presence, because we hypothesized that occupancy patterns might be indicative of underlying variation in plant defense or nutrition, and thus these populations could provide insight into aspects of host variation that are most important for *L*. *melissa*. We characterize intraspecific plant variation in terms of nutrition (measured as protein content) and phytochemistry. Additionally, given the likelihood of genetic variation among these focal alfalfa populations, we describe population genetic structure both to understand the degree of variation present within and among focal populations, and to explore the potential link between genetic differentiation and phytochemical divergence. We use these data to ask to what extent alfalfa populations differ in their effect on *L*. *melissa* larval performance and oviposition preference, and to what extent any observed differences (in performance or preference) can be explained by variation in host phytochemistry, protein content, or population genetic structure. Finally, we explore correlations between population genetic structure (of alfalfa), phytochemistry, and protein content, and discuss how observed patterns of intraspecific plant variation and herbivore responses might underlie historical patterns of *L*. *melissa* occupancy in alfalfa patches.

## Methods

Seven populations of alfalfa (*Medicago sativa*) from northwestern Nevada and California (Fig 1, S1 Table) were chosen, all occurring in disturbed areas, primarily along roadsides. Alfalfa populations differed in utilization by *Lycaeides melissa*, with some populations having no record of colonization and others having continuous presence of *L*. *melissa* for over a decade (MLF pers. observation). Recent absence of *L*. *melissa* at uncolonized populations was confirmed through observations conducted during the flight window (several trips to each site were made annually during the spring and summer months, May–August) from 2010–2013. Colonized alfalfa populations were located at Beckwourth Pass, CA (BWP); Verdi, NV (VUH); and Gardnerville, NV (GVL). Uncolonized populations (prefixed by the letter A) were located in west Reno, NV (AWFS); Fallon, NV (AFAL); and in the Central Valley of California north of Davis, CA (APLL). No permits were needed for the collection of alfalfa or *L*. *melissa* at these locations. Host use by *L*. *melissa* on alfalfa at Star Creek Canyon, NV (SCC) is probable but unconfirmed: most *L*. *melissa* individuals at this location were found near the native *Lupinus argenteus*, but individuals have also been observed in the vicinity of a small patch of roadside alfalfa. A single alfalfa population (APLL) extra-limital to the range of *L*. *melissa* was included in this study as a potential contrast to the northwestern Nevada populations. APLL is located approximately 100 miles from VUH, the closest Nevada population, on the other side of the Sierra Nevada mountain range (Fig 1). Plant foliage used for preference and performance assays was haphazardly collected from plants located throughout each focal alfalfa population (approximately 15 plants were sampled during each tissue collection event; only mature plants were sampled, avoiding seedlings). Because the presence of flowers may influence butterfly preference and larval performance, only foliar tissue was used in preference and performance assays. All alfalfa populations surveyed were roughly equivalent in terms of phenology (a majority of individuals were flowering whenever foliage was collected).

### Larval performance assay

Caterpillars used for the performance assay were obtained from eggs laid in July 2013 by females taken from Silver Lake, NV (SLA, Fig 1). These females utilize *Astragalus canadensis* (Fabaceae). Alfalfa does not occur at Silver Lake, and because *L*. *melissa* tend to be very localized in their movements [45], the females collected were unlikely to have previously encountered alfalfa. Females were placed in oviposition arenas (described below) containing only *A*. *canadensis*, and eggs were collected after two days and pooled. Within hours of hatching, caterpillars were placed singly in 20 x 90 mm petri dishes containing alfalfa from one of the seven source populations (Fig 1). Caterpillars were kept under lamps at room temperature (approximately 20°–23°C) and allowed to eat *ad libitum*, with leaves replaced at the first sign of wilting. Approximately sixty larvae were reared on each alfalfa population (n = 61 to 63, depending on the population).

Given the geographic distances separating alfalfa locations, it was not logistically feasible to keep all caterpillars supplied with sufficient fresh foliage to complete development. Therefore, most caterpillars were sacrificed at 14 days (after recording mass and survival), approximately halfway between egg hatch and adult eclosion. Caterpillars being fed alfalfa from three locations (VUH, AWFS, and APPL) were reared to adults, then sexed and weighed. These three locations were chosen because two of them (VUH and AWFS) are close to each other (~8.5 km apart) and close to the research laboratory at the University of Nevada, Reno, and initial results (after 14 days) from the third (APPL; which was extralimital to the range of *L*. *melissa*) suggested that it was potentially an informative, extreme contrast to the other two populations. All mass measurements were taken with a Mettler-Toledo XP26 microbalance to the nearest microgram.

### Oviposition preference assay

Oviposition preference of adult female butterflies was assayed as per [35], with females taken from either Silver Lake, NV (SLA) or Verdi, NV (VUH). As mentioned above, females from SLA are very unlikely to have encountered *M*. *sativa* prior to capture. Female *L*. *melissa* from VUH use alfalfa, and no known native host plants occur at this location. Females were placed in oviposition arenas containing alfalfa foliage from two out of the three sources that were used in the full-development rearing described above; specifically, either AWFS and APLL, or AWFS and VUH were paired in each oviposition arena. As a negative control, leaves from *Lotus nevadensis* were also included in each arena. *L*. *nevadensis* is a Fabaceous plant that *L*. *melissa* does not consume in the wild, but can subsist upon [34] *L*. *nevadensis* was collected near Yuba Gap, CA. These choice tests using wild-caught females are efficient and effective: they provide results that are similar to no-choice tests, and similar to results from lab-reared females [31].

Tests were conducted using plastic cups (12 cm x 9.5 cm) as oviposition arenas, with each cup containing three different branches (one branch each from the two alfalfa populations being compared, and the negative control) that the female could choose between. Branch stems protruded from small holes in the bottom of each cup. A second cup was filled with water and placed underneath the branch-containing cup, with stems submerged in water. Female butterflies were sealed inside these cups with fine mesh, and mesh was misted every two to four hours with tap water from 10 a.m. to 5 p.m. and swabbed with fruit punch Gatorade^®^ twice daily as a nectar substitute [35]. Oviposition arenas were kept outside in dappled shade on the University of Nevada, Reno campus at ambient temperature (approximately 20°–30°C). Assays were conducted from August 19, 2013 through September 5, 2013. After 48 hours within the arena, females were removed and the number of eggs laid on each plant was counted.

### Analyses of preference and performance data

Preference data were analyzed in a hierarchical Bayesian framework using the bayesPref package [46] in the R computing environment [47]. In contrast to a frequentist analysis of preference data that provides information regarding the rejection of a null of no difference in preference, this approach estimates preference for each of the different hosts (along with 95% equal-tail probability intervals [ETPIs]). Models employ a Markov chain Monte Carlo (MCMC) approach to characterize posterior probability distributions describing female preference for a plant at two hierarchies, that of the individual and the population (from which the female was drawn). For a full description of the form of the likelihood function and conditional priors for individual preference see [46]. Models were run for 20,000 MCMC iterations, with a 5,000 iteration burn-in, and output was examined to ensure adequate mixing. Larval survival was also analyzed with this methodology using counts of surviving individuals on different alfalfa sources as data (5,000 iterations with 1,000 iteration burn-in). This allowed us to estimate the probability of survival on a particular alfalfa source (in the same way preference for a particular alfalfa source is modeled with counts of eggs from preference experiments). Means of samples characterizing posterior distributions were used as point estimates for population level preference or survival. ETPIs of posterior probability distributions were examined for overlap in a pairwise fashion to determine differences in preference and survival among alfalfa populations. If ETPIs did not overlap for a given pairwise comparison, then preference or survival was inferred to differ between alfalfa populations. Finally, a Bayesian analysis of variance was used to determine differences in larval weight between populations [48]. Deflections from the mean for each population were sampled from normal distributions with a mean of zero and precisions were independently modeled for each population from folded t distributions (μ = 0, τ = 0.001). The grand mean was modeled from a normal distribution centered at zero with precision 0.001. Within-group variation was modeled independently to account for non-homogeneous variances among groups by sampling from a gamma distribution with shape and rate parameters of one. The model was run for 1,000,000 MCMC iterations divided between three chains with a burn-in of 5,000 iterations and a thinning rate of 1/100. ETPIs of posterior probability distributions of mean larval mass estimates were examined for overlap among populations as described above.

### Phytochemical analysis

Foliar tissue from 19–20 individuals from each alfalfa population was collected in late August 2013 (while larval performance experiments were ongoing), stored in paper bags, and frozen at -20°C until extraction. Plants selected were mature and collected haphazardly from throughout each population; the newest growth was avoided in phytochemical analyses. Approximately 150 mg of foliar tissue was extracted twice using a 70% meOH solution followed by 20 minutes of sonication. Samples were dried under reduced pressure, resuspended in 1 ml meOH, and passed through a 0.45 μm filter into an autosampler vial for high-performance liquid chromatography (HPLC) analysis. HPLC analyses were performed using a Waters Alliance HPLC system with a 2996 diode array detector and Empower Pro Software. Each injection was 10 μl eluted on a gradient (90:10 (water:acetonitrile), reaching 60:40 at 40 minutes and 5:95 at 60 minutes) at the rate of 1 ml/min on a Symmetry C-18 reverse phase column (3.5 μm, 4.6 x 75 mm) (Waters Corp.). Phytochemical variation was characterized by retention time and UV absorbance between 230 and 400 nm. This restricted our characterization of compounds to those recoverable by our extraction and HPLC protocol, and those with chromophores for UV absorption (usually those with double bonds), thereby providing a fingerprint of plant phytochemistry based on anonymous compounds. Many saponins (which are compounds known to occur within *M*. *sativa*) do not possess chromophores and consequently our method is not sensitive to these compounds. However, there are some saponins detectable via UV spectroscopy; additionally, our method should recover members of many other compound classes reported from *M*. *sativa* including flavonoids [49] and phytoestrogens [50]. Data were standardized by dry weight and Hellinger-transformed. This transformation is recommended prior to ordination of datasets containing many zeros, as was the case for our data [51].

Spectral data for all samples were ordinated using non-metric multidimensional scaling (NMDS) on a Manhattan distance matrix created using the vegan package in R (version 2.2 [52]). Examination of stress scree plots showed that an ordination across five dimensions provided the best compromise between stress reduction and complexity (with stress at 10.5). Linear discriminant analysis (LDA) was also employed using the MASS package (version 7.3 [53]) in R to explore phytochemical differences among alfalfa populations. LDA seeks to find the linear combination of predictor variables that best separate data by group, based on a pre-defined set of groups. We employed this approach to determine if phytochemical differences among alfalfa populations could predict *L*. *melissa* colonization of those populations. Also, we tested if observed phytochemical differences among populations could predict concomitant variation in larval performance.

To test if phytochemistry could predict *L*. *melissa* occupancy of an alfalfa population we first constructed a model trained using a randomly selected subset of the phytochemistry data (n = 90, for each iteration), and examined how well this model could correctly assign colonization status to validation data (n = 45, for each iteration). This process was repeated 10,000 times and the proportion of correct assignments was extracted at each iteration and tabulated. The mean of this vector of results was used as an estimate of model success. For this analysis we only used data for the 28 compounds that occurred across all alfalfa populations, as linear separation often occurred when using the whole dataset that included many rare compounds. We used Monte Carlo simulations to confirm that the results of the LDA would not be expected given a random distribution of chemotypes among populations, or a random distribution of compound concentrations among individuals (see S1 Appendix for details [54]).

### Exploring phytochemical diversity

Phytochemical variation within and between alfalfa populations was also examined using the complexity-as-diversity approach as described by [55]. This approach extends the use of numbers equivalents [56, 57] to phenotypic complexity using (Eq. 1 in [57]) Dq= (∑i=1spiq)1/(1−q) where the diversity (^*q*^*D*) represents the effective number of distinct phenotypes in a group, the diversity order *q* influences how sensitive *D* is to rare compounds, and *p* is the proportion of the data composed of the *i*-th compound (out of *s* total compounds). When *q* = 0, all compounds are weighted equally (i.e., chemical richness). When *q* = 1, compounds are weighted by their relative abundance. As *q* increases, the relative importance of rare compounds is reduced. Diversity profile plots across orders of *q* were used to assess the importance of rare and common compounds in determining the number of distinct chemical phenotypes (see [55]). This approach was taken at three nested hierarchical levels: plant, population, and global. At the within-population level, β-diversity is the effective number of distinct chemical phenotypes among plants (chemotypes), whereas at the among-population level, β-diversity is the effective number of distinct chemical phenotypes among populations. Diversity equivalencies were calculated using the vegetarian and hierDiversity packages in R [58, 59].

Finally, phytochemical covariance matrices were constructed using data specific to each population. These matrices were correlated with site-specific genetic covariance matrices (described below) and the significance of correlations was tested with Mantel tests (1,000 permutations; vegan package in R) to determine the extent to which differences between plant genotypes could explain phytochemical differences between those same individuals.

### Foliar tissue total protein determination

A Bradford assay [60] was used to ascertain the protein content of foliar tissue taken from the plants used in the phytochemistry assay (the same 19–20 individuals per population). See supplemental methods for a full description of protocol used (S1 Appendix). Prior to statistical analyses, absorbency data were standardized by sample mass. Site-specific distance matrices (Euclidean) of absorbencies were correlated with genetic and phytochemical distance matrices to test for relationships between these three axes of host variation at each alfalfa population (significance of correlations was determined using Mantel tests).

### Alfalfa population genetics

DNA was isolated and purified from desiccated (oven dried) leaf tissue sampled from 132 alfalfa plants using Qiagen's DNAeasy 96 Plant Kit (Qiagen Inc.). DNA was taken from the plants used in the phytochemistry and protein assays described above, however, DNA was only taken from individuals belonging to five of our seven focal populations. DNA was not successfully extracted from APLL and BWP samples; insufficient yield in these cases likely resulted from compromised DNA quality associated with drying. We generated DNA fragment libraries for genotyping-by-sequencing using our established protocol [61, 62, 63]. For details of our library preparation, sequencing, and bioinformatics protocol see supplemental methods. Briefly, genomic DNA was first enzymatically digested with the restriction enzymes EcoRI and MseI and double-stranded adaptor oligonucleotides ligated onto the digested DNA fragments. These adaptors included 8–10 base pair (bp) barcode sequences that were used to match sequences to individual plants. Fragment libraries were amplified using PCR and size-selected to fragments 250 and 350 bps in length using a BluePippin (Sage Science). Libraries were sequenced on an Illumina HiSeq 2500 (one lane, 1 x 100 bp reads) at the University of Texas Genome Sequence and Analysis Facility.

Sequences were aligned (236 million DNA sequences total) to a draft genome that we generated from the diploid progenitor of alfalfa (total scaffold length = 673 Mbp, N50 scaffold size = 37 kbp, number of scaffolds = 41319; we will more fully describe this genome sequence in a future publication, [64]). We then used the Unified Genotyper in GATK [65] to identify variable nucleotide positions and calculate genotype likelihoods for each individual and variable position (this is a Bayesian genotype and variant caller). We assumed a ploidy of four as alfalfa is a tetraploid. We set the minimum base quality to 20 and set the prior expectation for heterozygosity to 0.001. A custom Perl script was then used to filter the initial set of variants to those that met our quality criteria (see supplemental methods). Seventy-one plants (five populations) and 16,920 single nucleotide variants (SNVs) were retained for population genetic analysis.

We estimated the posterior probabilities of each genotype for each individual at each locus using a Bayesian approach. To do this we took the genotype likelihoods from GATK and multiplied them by the prior probabilities of each genotype assuming Hardy-Weinberg genotype frequencies and a maximum likelihood estimate of global non-reference allele frequency, which was also obtained using GATK. We then took the mean of the posterior distribution for each locus and individual as the genotype estimate for downstream analysis (this value is between zero and four, is not constrained to be an integer, and is an estimate of the number of non-reference allele copies at a locus). This general approach, which has been used previously [33, 63] allowed us to make better use of the information in low to moderate coverage population genomic data than if we had simply called genotypes directly from the sequence data for each individual.

We used several methods to quantify and summarize patterns of genetic variation within and among populations. We generated a genetic covariance matrix where each element in the matrix measured the genetic similarity (covariance in genotypes) for a pair of individuals. We then used principal components analysis (PCA) to visualize patterns of genetic similarity based on this matrix. Next, to quantify genetic variation within populations, we calculated the average genetic variance (1 − [p^2^ + (1 − p)^2^]) and the variance in PC 1 and 2 scores. Genome-average pairwise and global Fst values were then estimated from the sample allele frequencies to assess the extent of population genetic structure. Finally, we tested for a positive correlation between genome-average Fst and the geographic distance between pairs of populations to determine whether alfalfa showed signs of isolation-by-distance (a Mantel test with 1,000 permutations was used to test whether the observed correlation was significantly different from zero at α = 0.05).

## Results

### Larval performance

**Fig 2:**
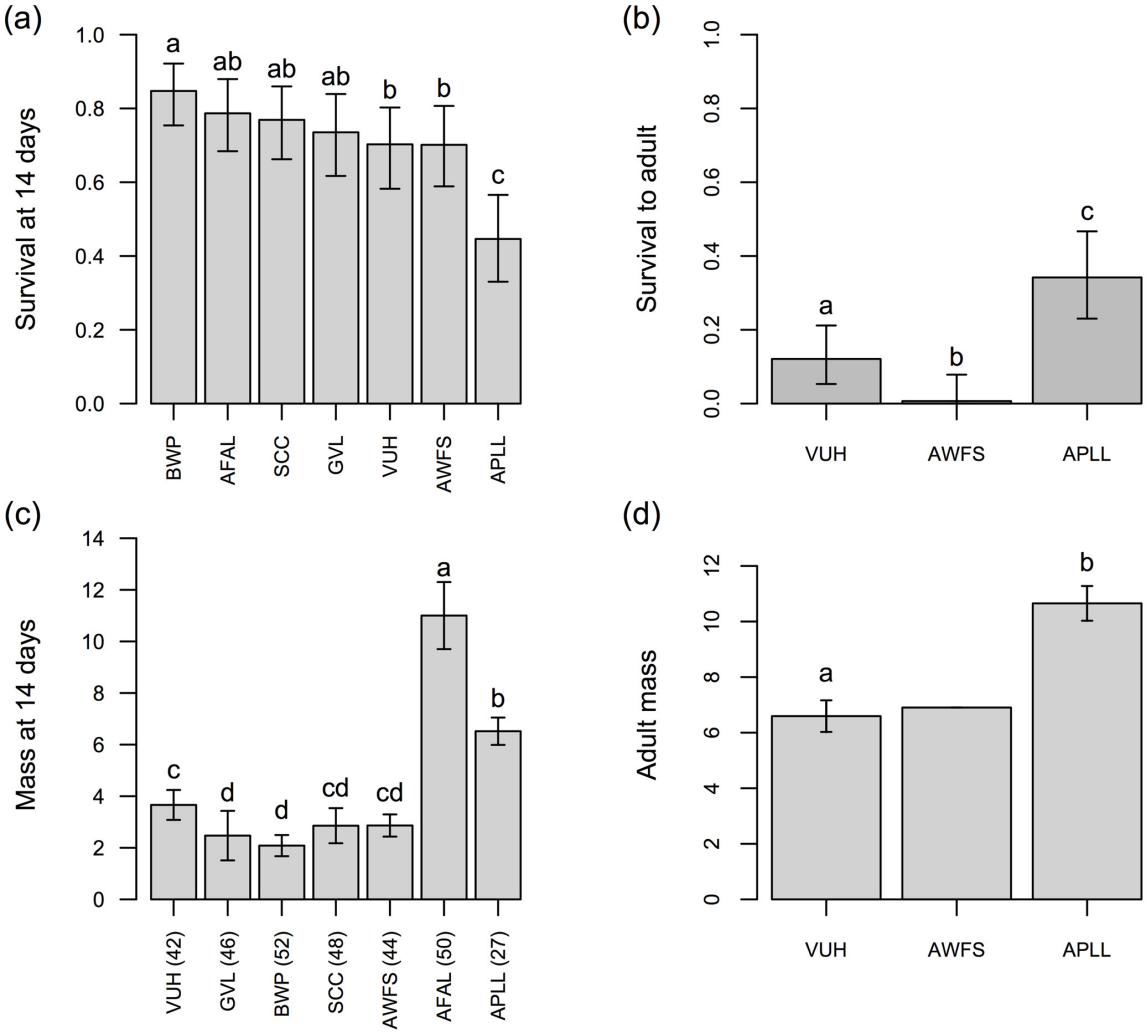
Results from caterpillar performance trials: survival (a & b), and mass (c & d). Performance in (a) and (c) are across all alfalfa source populations through 14 days of development (>60 larvae reared on each alfalfa population); performance in (b) and (d) involve caterpillars reared to adults on a subset of alfalfa sources (number of surviving, weighed larvae in parentheses). Bars in (a) and (b) are 95% equal-tail probability intervals (ETPIs) of survival rate estimates; bars in (c) and (d) are 95% ETPIs of estimates of mean mass. AWFS in panel (d) was not included in analyses because we only have mass from a single individual (no other larvae survived on alfalfa from this population).

A total of 434 caterpillars were fed alfalfa from seven different alfalfa source locations. Survival in the first half of development was quite variable across populations, ranging from less than 50% to greater than 80% (Fig 2a). Interestingly, the alfalfa source (APLL) conferring the lowest larval survival in the first 14 days supported the highest survival (among a subset of three alfalfa sources) across the entire course of development (Fig 2b). Mass was also variable among treatment groups (Fig 2c), with individuals consuming alfalfa rom two of the sources (AFAL and APLL) being two or more times greater in mass than the individuals reared on the other alfalfa sources. The consumption of APLL alfalfa was associated with the largest butterflies at the end of development (Fig 2d), and fastest time to eclosion as compared with VUH and AWFS alfalfa (Wilcoxon rank sum test, p < 0.01) (S1 Fig).

### Oviposition preference

**Fig 3:**
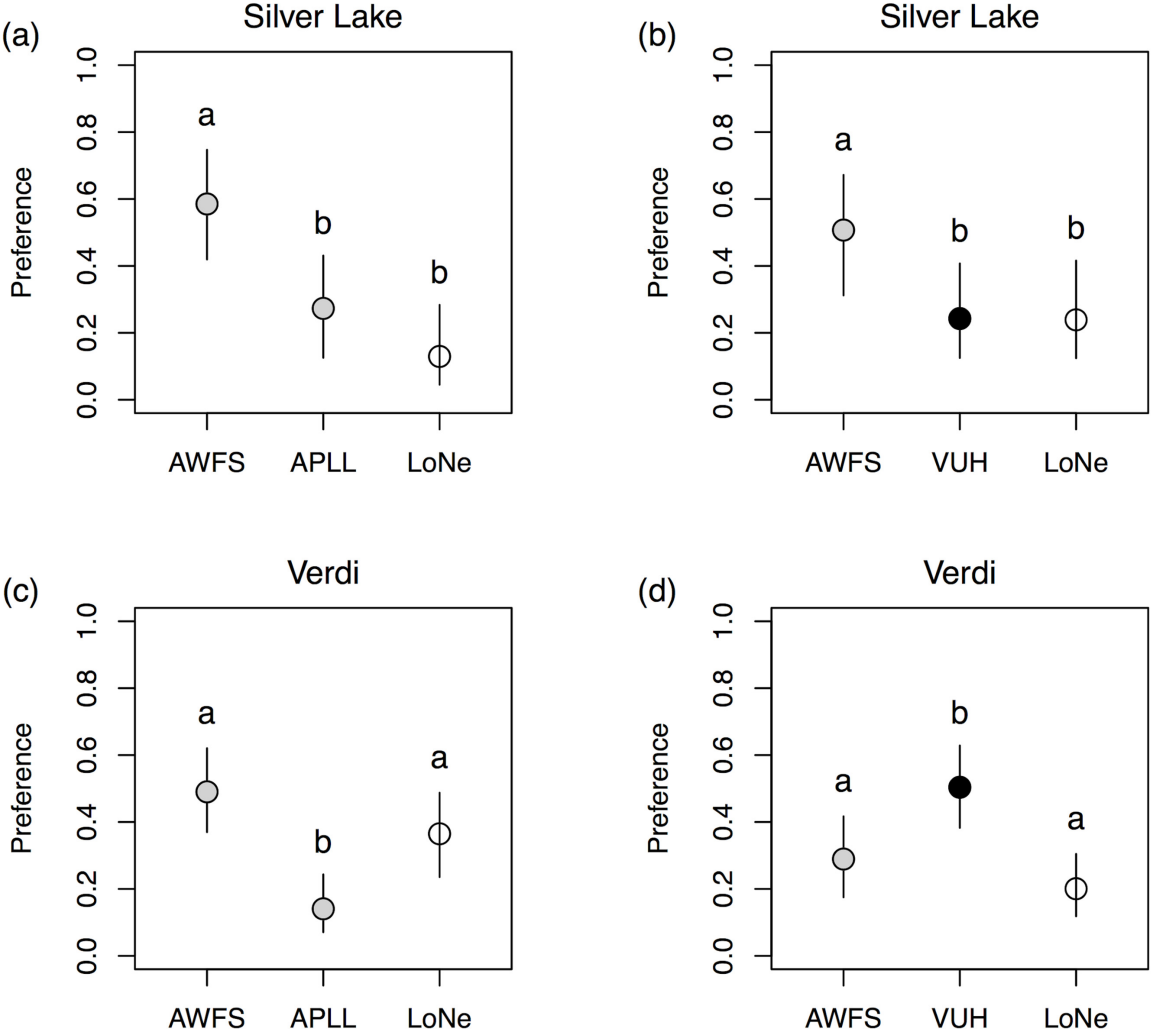
Preference of Silver Lake (a & b) and Verdi females (c & d) for different combinations of alfalfa sources (gray symbols) and a negative control (open symbol). Silver Lake (SLA) females were from a native-host population (no previous exposure to alfalfa), and Verdi females were from an alfalfa-feeding population (VUH). Bars denote 95% equal-tail probability intervals for population preference and symbols indicate the mean of posterior probability distribution. Different letters denote non-overlapping probability intervals.

We challenged females presumed naïve to alfalfa (females were from SLA, where alfalfa is not known to occur) and females associated in the wild with alfalfa (from VUH) with plants from either VUH and AWFS, or from AWFS and APLL, the three alfalfa sources used in extended larval rearing (see S2 Table for the number of females assayed and other details). Females from SLA showed greater preference for AWFS alfalfa in both trials, compared to APLL and VUH alfalfa and the negative control (Fig 3a and 3b). The behavior of alfalfa-associated females (from VUH) was more complex: they discriminated between AWFS and APLL (Fig 3c), but then preferred alfalfa from their home location when given a choice between VUH and AWFS (Fig 3d). In one of the experiments (VUH females choosing between AWFS and APLL; Fig 3c), the negative control received more eggs than one of the alfalfa sources (APLL). This willingness to lay eggs on the negative control is consistent with behavior previously observed in females from this area [35].

### Protein content

**Fig 4:**
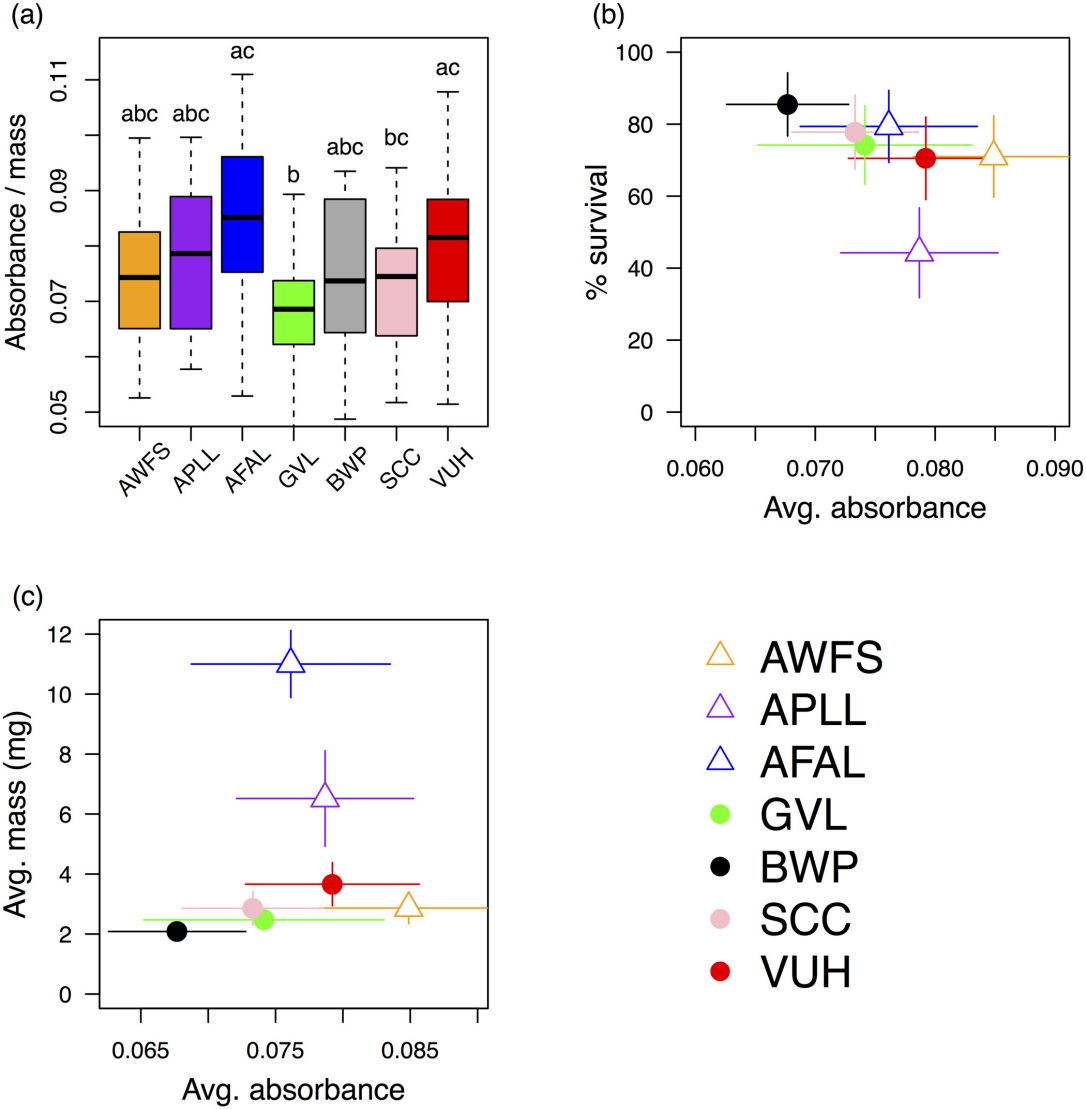
Results from Bradford assay of foliar protein in seven populations of alfalfa. (a) Absorbance (standardized by mass) by population, with significant differences denoted via superscripts (Kruskal-Wallis). Absorbance is directly proportional to protein content. (b & c) Relationships between protein concentration and larval survival and mass gain at 14 days were not significant. Lines denote standard errors of mean estimates.

Variation in total protein content was observed among surveyed alfalfa populations (Kruskal-Wallis test, χ^2^ = 17.7, df = 6, p < 0.01; a rank-based test was used because of non-normality of data) (Fig 4a). Plants from VUH and AFAL had significantly higher protein content compared with those from GVL (post-hoc multiple comparison test after Kruskal-Wallis, p ≤ 0.05). We failed to detect a significant correlation between protein content at the population level and larval weight gain or survival: Spearman’s rank correlation, rho = 0.61, p = 0.16, and rho = -0.75, p = 0.07, for weight gain and survival respectively (Fig 4b and 4c). Neither did we detect significant correlations between distance matrices of protein content, phytochemistry, or genetic covariance (S3 Table).

### Phytochemistry

**Fig 5:**
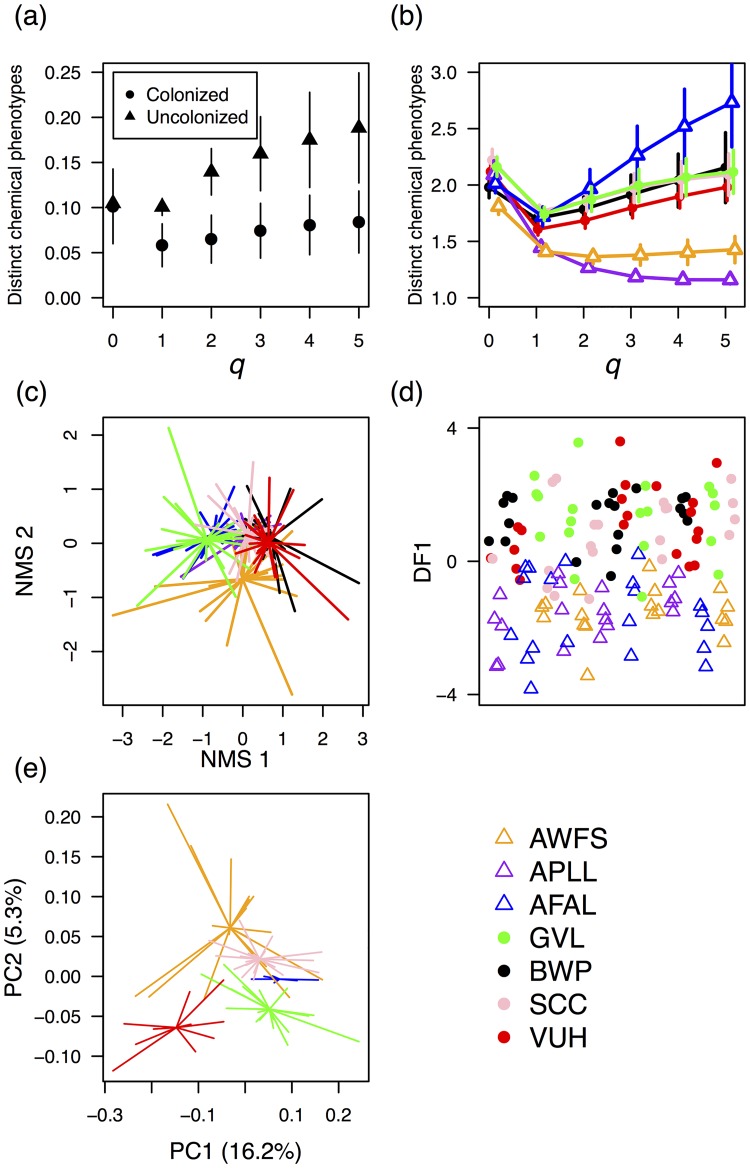
Phytochemistry and genetic structure of surveyed alfalfa populations. (a) Number of distinct chemical phenotypes for populations colonized by *L*. *melissa* (circles), and uncolonized populations (triangles). Higher values of q reflect higher order diversity equivalents. Abundant compounds are more heavily weighted at values of q > 1. Underlying phytochemical data was collected via HPLC. (b) Uncolonized populations were phytochemically dissimilar, but typically had low within-population phytochemical variation. On the other hand, colonized populations were phytochemically similar, despite the fact that these populations tended to exhibit more within-population phytochemical variation than uncolonized populations (also see S2 Fig). (c) NMDS of phytochemical data showed overall similarity in phytochemistry across surveyed alfalfa populations. (d) However, LDA showed that important phytochemical variation did exist, and could well separate populations differing in colonization by *L*. *melissa*. Each point represents an individual plant. Values plotted were calculated by substituting each datum into the discriminant function. The x-axis is not labeled because it serves only to spread samples for visualization. (e) PCA of genetic covariance matrix constructed using ~17,000 SNVs.

Our HPLC protocol resulted in data for 49 compounds. Of these, 28 compounds occurred in all alfalfa populations, five occurred only in populations colonized by *L*. *melissa*, and two occurred only in uncolonized populations. Consequently, phytochemical α-diversity was very similar across populations (S2 Fig). Calculation of phytochemical β-diversity across all populations suggested few distinct chemical phenotypes were present (~1.2 chemotypes; S2 Fig). However, when grouping populations in terms of *L*. *melissa* colonization, we found that colonized populations exhibited less among-population chemical heterogeneity (Fig 5a), but had similar levels of within-population phytochemical heterogeneity, which also tended to be higher than in uncolonized populations (Fig 5b). AFAL was an exception to this pattern, as it exhibited high within-population phytochemical variation, but was uncolonized by *L*. *melissa* (Fig 5b).

NMDS ordination suggested considerable overlap in phytochemistry among populations (Fig 5c, S3 Fig). However, LDA demonstrated that important phytochemical variation among populations did exist. Indeed, phytochemical differences between colonized and uncolonized populations allowed for near complete separation by LDA (Fig 5d). This result held both when analyzing data for all 49 compounds, and when analysis was limited to those compounds occurring at all alfalfa populations (28 compounds) (S4 Fig). When limiting the analysis to compounds occurring at all populations, the mean estimate of the ability of LDA to successfully predict colonization status was 67% (95% confidence intervals: 53%–80%). The predictive ability of the LDA was confirmed by comparison to output of simulated null models (see S1 Appendix). LDA also demonstrated differences in phytochemistry among all seven populations (S5 Fig). For this analysis, the top four functions output by LDA provided 28.4%, 21.2%, 18.1%, and 15.7% separation respectively (S5 Fig). However, while providing good separation of alfalfa populations in terms of phytochemistry, these discriminant functions did not predict larval performance (S5 Fig). Finally, we found little correlation between our measures of genetic and phytochemical variation among individual plants. Genetic covariance and phytochemical covariance were not correlated at any population, save for a weakly-positive signal at GVL (Mantel tests, S4 Table).

### Population structure

A total of 16,920 single nucleotide variants were identified and used to construct a genetic covariance matrix, which was analyzed via PCA. PCs 1 and 2 explained 16.2% and 5.3% of the variation in pairwise genetic similarities among individuals (Fig 5e). VUH and GVL differed most with respect to PC1, whereas PC2 separated these populations from AWFS. The AWFS population was notably more variable with respect to both PC1 (var = 0.011) and PC2 (var = 0.0052) scores (var PC1: AFAL = 0.003, GVL = 0.006, SCC = 0.005, VUH = 0.008; var PC2: AFAL < 0.0001, GVL = 0.0008, SCC = 0.0004, VUH = 0.0011). All five populations exhibited similar levels of genetic variance (0.27–0.29). Overall, little genetic variation was partitioned among populations (global Fst = 0.020, 95% bootstrap CIs 0.020–0.021). However, population pairs varied in the degree to which they differed from each other (mean pairwise Fst = 0.013, minimum = 0.008, maximum = 0.019) (S5 Table). Finally, we found no evidence that genetic differences were greater between more geographically distant populations (Mantel r = -0.463, p = 0.95).

## Discussion

For any pair of interacting organisms, the strength, and even the occurrence, of the interaction tends to be variable across the geographic ranges of the interacting species [66, 67]. Understanding this heterogeneity is important for predicting the ecological and evolutionary consequences of the interaction, but requires detailed empirical work on intra-specific variation that has not been performed for a great many pairs of interacting plants and insects in the wild (for examples of such work see [27, 68, 69, 70, 71, 72]). Here we examined a system in which the herbivore (*L*. *melissa*) has been well characterized for intra-specific variation in numerous traits but the plant had previously been treated as a single entity (i.e. without accounting for inter-population differences). We found inter-population differences with respect to three axes of plant variation (nutrition, phytochemistry, and genetic variation) that affect *L*. *melissa*. The plant traits of largest effect to *L*. *melissa* were not the same for adults and juveniles, which adds complexity to the task of predicting the outcome of this novel plant-insect interaction.

Not only did plant effects differ between adults and larvae, but there was also a reversal of relative host quality when comparing early instar survival with survival to adulthood. Alfalfa obtained from VUH supported higher larval survival for the first two weeks (>60% survival, Fig 2a) compared with alfalfa from APLL, which supported the worst larval survival over the same time period (<50% survival, Fig 2a). Conversely, when considering survival to adulthood, APLL supported the highest survivorship (~30% on APLL versus ~10% for VUH, Fig 2b). No such reversal of suitability was observed for mass gain: APLL supported some of the largest caterpillars at two weeks (second to AFAL) as well as the largest adult butterflies. Taken together, these findings suggest that APLL is the better host, yet APLL was always the least preferred substrate in the oviposition assays we conducted. This was true even when APLL was paired with the nearly-fatal foliage from AWFS (Fig 3). While a mismatch between preference and performance has been demonstrated numerous times in other systems, it has typically been investigated in the context of interspecific differences (e.g.[73, 74], but see [75]), rather than differences between plant populations or individuals [76]. Notably, despite the mismatch we observed, alfalfa-associated females chose optimally when presented with hosts from their area. Specifically, females from VUH preferred their natal host plants to hosts from the neighboring AWFS population (~8.5 km apart). This is consistent with a hypothesis of ongoing preference evolution in *L*. *melissa*, which has been suggested previously in a survey of preference in ten *L*. *melissa* populations [35].

While we discovered dramatic differences in the extent to which different alfalfa sources support larval development or elicit oviposition, linking population-specific variation in alfalfa nutrition, chemistry, or genetic variation to *L*. *melissa* colonization status remains challenging. However, phytochemical variation shows some promise for being a successful predictor of alfalfa occupation (Fig 5d), suggesting an important (albeit not yet understood) role of phytochemistry in determining the extent to which host populations can support *L*. *melissa* colonization and persistence. Furthermore, we found that uncolonized populations are phytochemically different from one another (Fig 5a), yet tend to have (in two out of three cases) lower within-population phytochemical diversity compared with colonized populations (Fig 5b). Although more populations need to be studied to confirm this possibility, it might be the case that more phytochemically diverse alfalfa populations are older, and have thus had a longer time to become colonized by *L*. *melissa*. Unfortunately, we have no way of gauging alfalfa population age and cannot test this possibility. Alternatively, a more direct link between phytochemical diversity and colonization is possible. If individual females vary in terms of preference for a given host phenotype [18, 77, 78], then immigrating females would be more likely to encounter a plant deemed acceptable for oviposition in a phenotypically-complex host patch. The possible importance of within-patch variation in host phenotype is also interesting in light of the poor correlation we observed between genetic and phytochemical differences among individuals. The lack of a genetic-phytochemistry correlation suggests that plasticity might play a role in maintaining the intra-population variation we observed in occupied alfalfa populations [79]. With respect to plasticity of chemical phenotypes, it should also be noted that *L*. *melissa* caterpillars tend to be at very low densities relative to other herbivores (JGH & MLF pers. observations), thus we consider it unlikely that *L*. *melissa* herbivory per se drives phytochemical diversity in colonized patches through induction of host defenses. Finally, the inference of plasticity should be tempered for now by the resolution of our genetic analyses. Although we assayed thousands of genetic regions (more than 16,000 SNPs), analyses focused on overall similarity and thus could obscure important genetic variants that influence phytochemistry.

Beyond the overall differences between occupied and unoccupied locations (Fig 5d), other aspects of plant phenotype (such as nutritional differences) do not appear to explain colonization status. Nevertheless, the case of VUH and AWFS alfalfa is intriguing: they are geographically proximate, though the unoccupied location (AWFS) is nearly-lethal to caterpillars and is not preferred by ovipositing alfalfa-associated females (from VUH). Given the lack of significant differences in foliar protein, we suspect differences in plant suitability are due to the phytochemical separation between these populations (S5 Fig). The VUH versus AWFS comparison raises the possibility that a larger sample of paired alfalfa sites (occupied and unoccupied) could reveal differences informative to *L*. *melissa* population persistence (and provide the statistical power to identify compounds of large ecological effect). Future studies should also, of course, attempt to measure other factors relevant to population colonization and persistence, including geographic barriers to *L*. *melissa* dispersal, the role of natural enemies, and the age of alfalfa populations.

In summary, we found dramatic consequences of intraspecific variation among alfalfa populations for *L*. *melissa* larval performance and oviposition preference. This result provides an important foundation for our understanding of metapopulation dynamics in this system. In a population genomic survey of hundreds of *L*. *melissa* individuals throughout the Great Basin, the majority could be assigned to their population of origin based on their genotypes [63]. Thus, movement across the landscape appears to be infrequent, and new host patches are probably colonized by only a few dispersing individuals. Given the variation in alfalfa patch suitability that we describe here, dispersal of *L*. *melissa* may be further constrained because not all emigrating *L*. *melissa* adults will encounter alfalfa patches that can support larval development. These ecological filters suggest a mechanism explaining why novel host use by *L*. *melissa* in the Great Basin is characterized by the founder bottlenecks uncovered by [63].

In a study of the genetic architecture of host use by *L*. *melissa*, [33] found a large number of genetic regions with conditionally-neutral effects across hosts; in other words, some loci have alleles that affect performance on one host, but have little or no effect on another host, and vice versa. Given that conditionally-neutral genetic architecture, [33] hypothesized that, in this system, drift could promote the evolution of specialization by leading to the loss of alleles associated with an ancestral host. This route to specialization would be most likely in a situation where founder bottlenecks are severe, which could be promoted by large differences in suitability among host patches, such as those documented in the current study. Finally, our results reaffirm the importance of considering variation among conspecifics in the study of interactions, and suggest new lines of questioning regarding how habitat suitability may be influenced by variation in phytochemistry and host nutrition.

## Supporting Information

S1 Appendix Additional methods. (DOCX)

S1 Fig Development time. Days to eclosion significantly differed between *L*. *melissa* larvae reared on alfalfa sourced from APLL and VUH (Wilcoxon rank sum test, p < 0.01). Days to eclosion reflects time elapsed from hatching of first instar through to eclosion of adult butterflies. Sample sizes shown reflect those butterflies that survived out of the initial ~60 larvae reared on each host population).
(DOCX)

S2 Fig Chemical phenotypic complexity. Diversity equivalencies calculated using phytochemistry data from each alfalfa population. Equivalencies were calculated using increasing values of the q parameter to explore how they might change as the more abundant compounds were more heavily weighted in calculations. α-diversity reflects chemical richness within an individual plant, β-diversity the distinct number of chemical phenotypes among plants or populations (chemotypes), and γ-diversity total phytochemical diversity within a population. Standard errors were calculated using 1,000 randomizations.
(DOCX)

S3 Fig NMDS ordination of phytochemical data. Non-metric multidimensional scaling (NMDS) ordination of phytochemical data obtained from seven populations of alfalfa (see Fig 1 in main text for population locations). Data consisted of peak intensity information for 49 compounds as characterized through HPLC analysis. The average value of a population on each NMDS dimension was calculated and plotted, lines emanating from this centroid extend to each datum (individual plant). Ordination demonstrated a high degree of phytochemical similarity between populations.
(DOCX)

S4 Fig Phytochemical separation between alfalfa populations differing in *L*. *melissa* colonization. Phytochemical separation between alfalfa populations differing in *L*. *melissa* colonization. The top row depicts results of linear discriminant analysis. In the top left panel all 49 compounds characterized via HPLC were analyzed, while in the top right panel only those compounds occurring at all alfalfa populations were analyzed. Points are individual plants, triangles are used for alfalfa populations uncolonized by *L*. *melissa*, circles for colonized populations. The bottom left panel shows differences in compound concentration between colonized and uncolonized alfalfa populations. Differences in means shown reflect differences in mean peak intensity for a given compound. This frequency distribution only includes those compounds that occurred at all alfalfa populations analyzed.
(DOCX)

S5 Fig Results of linear discriminant analysis. Phytochemistry of surveyed alfalfa populations. The top row depicts results of linear discriminant analysis of 49 compounds characterized via HPLC (each combination of axes is a different combination of discriminant functions). Points are individual plants, triangles are used for alfalfa populations uncolonized by *L*. *melissa*, circles for colonized populations. Average values output by discriminant functions for a population are plotted by larval survival (row 2) and larval mass (row 3) to determine if the compounds that best differentiate alfalfa populations can predict the differences in larval performance, or colonization status, observed between those same populations. Discriminant functions proved poor predictors of larval performance.
(DOCX)

S1 Table Study location. Location and elevation of alfalfa (*Medicago sativa*) populations utilized for this study.
(DOCX)

S2 Table Oviposition assay details. Collection and sample size information for each oviposition assay performed. Each assay consisted of challenging a single female *L*. *melissa* butterfly with alfalfa from two populations and a negative control (*Lotus nevadensis*). All oviposition assays were conducted for 48 hours outdoors at ambient temperature. The two dates for Silver Lake were because inclement weather caused few eggs to be laid during the first assay of SLA females’ preference between AWFS and APPL. Consequently, two days later this assay was performed again using fresh females and data from the two assays were pooled.
(DOCX)

S3 Table Correlation of protein content with genetics and phytochemistry. Results from multiple mantel tests correlating a distance matrix of protein content with both a phytochemical distance matrix, and a genetic covariance matrix generated from alfalfa (*Medicago sativa*) individuals sourced from five populations (see main text for locations). Protein data was generated via a Bradford assay (absorbance/divided by mass). Phytochemistry data consisted of a matrix of peak intensity for 49 compounds (HPLC data again standardized by dry weight); and, genetic data consisted of a pairwise genetic covariance matrix (generated using 16,920 SNVs). All data were converted to distance matrices using a Euclidean distance measure, then analyzed with a multiple mantel test (1,000 permutations). Correlation coefficients using both Pearson’s product-moment correlation and Spearman’s rank correlation are given along with corresponding p values.
(DOCX)

S4 Table Correlation of genetics with phytochemistry. Results from multiple Mantel test correlating phytochemical and genetic distance matrices generated from alfalfa (*Medicago sativa*) individuals sourced from five populations (see main text for locations). Phytochemistry data consisted of a matrix of peak intensity for 49 compounds (HPLC data standardized by dry weight); and, genetic data consisted of a pairwise genetic covariance matrix (generated using 16,920 SNVs). Both matrices were converted to distance matrices using a Euclidean distance measure, then analyzed with a multiple mantel test (1,000 permutations). Correlation coefficients using both Pearson’s product-moment correlation and Spearman’s rank correlation are given along with corresponding p values.
(DOCX)

S5 Table Genetic structure in surveyed alfalfa populations. Pairwise Fst values for alfalfa (*Medicago sativa*) populations examined. Populations prefixed by “A” were not colonized by *L*. *melissa* See main text for analytical and sequencing details.
(DOCX)
