# Supplementary material for: The Many Dimensions of Diet Breadth: Phytochemical, Genetic, Behavioral, and Physiological Perspectives on the Interaction between a Native Herbivore and an Exotic Host
Source: PLoS One. 2016 Feb 2;11(2):e0147971. doi: 10.1371/journal.pone.0147971 (PMC4737494; doi:10.1371/journal.pone.0147971)
Supplement: S1 Appendix — (DOCX) [file pone.0147971.s001.docx]

**S1 Appendix: Additional methods**

*LDA simulation analysis methods*

Linear discriminant analysis (LDA) was employed using the MASS package (version 7.3, Venables and Ripley 2002) in R to explore phytochemical differences among alfalfa populations. LDA seeks to find the linear combination of predictor variables that best separate data by group, based on a pre-defined set of groups. We employed this approach to determine if phytochemical differences among alfalfa populations could predict *L. melissa* colonization of those populations (see main text for details).

Given the large number of predictor variables used in our LDA of phytochemistry (28 compounds) we used a Monte Carlo approach to confirm the adequate performance of the analysis. In other words, we wished to test if the ratio of the number of predictor variables to the number of measurements of the independent variable in our data perforce led to generation of well-performing, discriminatory functions. Consequently, we constructed two null models to test the predictive power of our discriminant function above null expectations for a dataset such as ours.

The first null model tested if any two arbitrarily assigned groupings of our data could be well predicted by LDA (a random distribution of chemotypes among populations). For this null model, we randomly assigned colonization status to each of our samples (individuals) and built a discriminant function using these data. As per above, we repeated this process 10,000 times and saved the proportion of correct assignments (of colonization status) generated at each iteration. We considered the simulation to have outperformed the LDA when the proportion of correct assignments obtained in an iteration of the simulation was greater than the mean proportion of correct assignments obtained by the LDA on observed data (as per Gotelli and Ellison 2013). By counting the number of times the simulation outperformed the LDA of observed data, and dividing this count by the total number of iterations (10,000), we were able to calculate a p-value for how well our LDA performed above the null expectation as described via this simulation.

We also tested that the results of LDA on our data would not be expected given a random distribution of concentrations of compounds among samples. To test this possibility, we took the vector of observed concentrations for a given compound and randomly assigned, without replacement, values from this vector to each sample. We did this for each of the 28 compounds, and built a discriminatory function using these randomized data. Again, we trained and tested discriminant functions generated with these data 10,000 times, and saved the proportion of correct assignments generated at each iteration. As described above, we tested if the results output from this simulation where different from those output by our LDA of observed data.

*Results*

The mean estimate of the ability of LDA to successfully predict colonization status when data analyzed were limited to compounds common across all alfalfa populations was 67% (95% confidence intervals: 53%–80%; this is the mean percentage of correct assignments of validation data a colonization status across 10,000 trials, see methods in main text). This represents a significant outperformance of both null models that tested the veracity of the predictive power of the LDA given our data structure (p = 0.0257 for the simulation randomizing chemotype among groups; and p = 0.0112 for the simulation that randomized chemical concentrations among individuals).

*Details of Bradford assay*

A Bradford assay (Bradford 1976) was used to ascertain protein content of foliar tissue taken from the same individual plants used in the phytochemistry assay (19-20 individuals per population). Individual *M. sativa* plants were collected from each population in late August of 2013. Plants were stored dried and frozen at -20º C until analysis. Approximately 25 mg of foliar tissue from each individual was ground to a fine powder and weighed to the nearest thousandth of a milligram. Ground tissue was extracted in 1 ml of 0.1 M NaOH for thirty minutes and then centrifuged for 5 minutes at 5,000 rpm. Aliquots of the resulting supernatant were combined with BioRad reagent and assayed with a BioRad microplate reader set at 595 nm. Two foliar samples were assayed in triplicate from each individual plant and absorbencies of all six samples averaged. Prior to statistical analyses, absorbency data obtained for all samples were standardized by sample mass. A distance matrix (Euclidean) of absorbencies by individual was correlated with genetic and phytochemical distance matrices (significance tested with Mantel tests) to test for relationships between these three axes of host variation.

*Population genetics analysis details*

DNA was isolated and purified from desiccated (oven dried) leaf tissue sampled from 132 alfalfa plants using Qiagen's DNAeasy 96 Plant Kit (Qiagen Inc.). These plants were taken from five of our seven focal populations. DNA was not successfully extracted from APLL and BWP samples; insufficient yield in these cases likely resulted from compromised DNA quality associated with drying. We generated DNA fragment libraries for genotyping-by-sequencing using our established protocol (Gompert et al. 2012, Parchman et al. 2012, Gompert et al. 2014). Specifically, genomic DNA was first enzymatically digested with the restriction enzymes EcoRI and MseI. Double-stranded adaptor oligonucleotides were then ligated onto the sticky-ends of the digested DNA fragments. These adaptors included 8-10 base pair (bp) barcode sequences that were used to match sequences to individual plants. Fragment libraries were amplified using PCR and size-selected to fragments 250 and 350 bps in length using a BluePippin (Sage Science). Libraries were sequenced on an Illumina HiSeq 2500 (one lane, 1 x 100 base pair reads) at the University of Texas Genome Sequence and Analysis Facility.

We aligned the DNA sequences (236 million DNA sequences total) to a draft genome generated from the diploid progenitor of alfalfa (total scaffold length = 673 Mbp, N50 scaffold size = 37 kbp, number of scaffolds = 41319; we will more fully describe this genome sequence in a future publication). Sequences were aligned using the aln algorithm in bwa (Li and Durbin 2009) with a maximum of 5 mismatches, and a 20 bp seed with only two allowed mismatches in the seed. Bases with quality scores lower than 10 were excluded from the alignment. We used samtools to compress, sort and index the alignments. We then used the Unified Genotyper in GATK (DePristo et al. 2011) to identify variable nucleotide positions and calculate genotype likelihoods for each individual and variable position (this is a Bayesian genotype and variant caller). We assumed a ploidy of four as alfalfa is a tetraploid. We set the minimum base quality to 20 and set the prior expectation for heterozygosity to 0.001. A custom Perl script was then used to filter the initial set of variants. We retained those variants that met the following criteria: 2x minimum average coverage, eight or more sequences containing the non-reference allele, less than 10% of sequences spanning an insertion-deletion, no more than five mapping quality zero sequences, a minimum mapping quality of 30, at least one sequence in 72% of individuals, a maximum absolute value of the mapping quality rank sum test of two, a maximum absolute value of the base quality rank sum test of three, and a minimum ratio of variant confidence to non-reference sequence coverage of two. We also excluded alfalfa samples with mean sequence coverage less than 2x (i.e. we dropped low coverage nucleotide positions and low coverage individuals). 71 plants (five populations) and 16,920 single nucleotide variants (SNVs) were retained for population genetic analysis.

We estimated the posterior probabilities of each genotype for each individual at each locus using a Bayesian approach. To do this we took the genotype likelihoods from GATK and multiplied them by the prior probabilities of each genotype assuming Hardy-Weinberg genotype frequencies and a maximum likelihood estimate of global non-reference allele frequency, which was also obtained using GATK. We then took the mean of the posterior distribution for each locus and individual as the genotype estimate for downstream analysis (this value is between zero and four, is not constrained to be an integer, and is an estimate of the number of non-reference allele copies at a locus). This general approach, which we have used previously (e.g. Gompert et al. 2014, Gompert et al. 2015), allowed us to make better use of the information in low to moderate coverage population genomic data than we would have been able to if we had simply called genotypes directly from the sequence data for each individual.

We used several methods to quantify and summarize patterns of genetic variation within and among populations. We generated a genetic covariance matrix where each element in the matrix measured the genetic similarity (covariance in genotypes) for a pair of individuals. We then used principal components analysis (PCA) to visualize patterns of genetic similarity based on this matrix. Next, to quantify genetic variation within populations, we calculated the average genetic variance (1 - [p^2^ + (1 - p)^2^]) and variance in PC 1 and 2 scores. Genome-average pairwise and global Fst were then estimated from the sample allele frequencies to assess the extent of population genetic structure. Finally, we tested for a positive correlation between genome-average Fst and the geographic distance between pairs of populations to determine whether alfalfa showed signs of isolation-by-distance (a Mantel test with 1,000 permutations was used to test whether the observed correlation was significantly different from (α = 0.05) zero).
