# Supplementary material for: The Many Dimensions of Diet Breadth: Phytochemical, Genetic, Behavioral, and Physiological Perspectives on the Interaction between a Native Herbivore and an Exotic Host
Source: PLoS One. 2016 Feb 2;11(2):e0147971. doi: 10.1371/journal.pone.0147971 (PMC4737494; doi:10.1371/journal.pone.0147971)
Supplement: S1 Fig — Days to eclosion significantly differed between L. melissa larvae reared on alfalfa sourced from APLL and VUH (Wilcoxon rank sum test, p < 0.01). Days to eclosion reflects time elapsed from hatching of first instar through to eclosion of adult butterflies. Sample sizes shown reflect those butterflies that survived out of the initial ~60 larvae reared on each host population). (DOCX) [file pone.0147971.s002.docx]

S1 Figure. Days to eclosion significantly differed between *L. melissa* larvae reared on alfalfa sourced from APLL and VUH (Wilcoxon rank sum test, p < 0.01). Days to eclosion reflects time elapsed from hatching of first instar through to eclosion of adult butterflies. Sample sizes shown reflect those butterflies that survived out of the initial ~60 larvae reared on each host population).
