# Supplementary material for: The Many Dimensions of Diet Breadth: Phytochemical, Genetic, Behavioral, and Physiological Perspectives on the Interaction between a Native Herbivore and an Exotic Host
Source: PLoS One. 2016 Feb 2;11(2):e0147971. doi: 10.1371/journal.pone.0147971 (PMC4737494; doi:10.1371/journal.pone.0147971)
Supplement: S2 Fig — Diversity equivalencies calculated using phytochemistry data from each alfalfa population. Equivalencies were calculated using increasing values of the q parameter to explore how they might change as the more abundant compounds were more heavily weighted in calculations. α-diversity reflects chemical richness within an individual plant, β-diversity the distinct number of chemical phenotypes among plants or populations (chemotypes), and γ-diversity total phytochemical diversity within a population. Standard errors were calculated using 1,000 randomizations. (DOCX) [file pone.0147971.s003.docx]

S2 Figure. Diversity equivalencies calculated using phytochemistry data from each alfalfa population. Equivalencies were calculated using increasing values of the q parameter to explore how they might change as the more abundant compounds were more heavily weighted in calculations. α-diversity reflects chemical richness within an individual plant, β-diversity the distinct number of chemical phenotypes among plants or populations (chemotypes), and γ-diversity total phytochemical diversity within a population. Standard errors were calculated using 1,000 randomizations.
