# Supplementary material for: The Many Dimensions of Diet Breadth: Phytochemical, Genetic, Behavioral, and Physiological Perspectives on the Interaction between a Native Herbivore and an Exotic Host
Source: PLoS One. 2016 Feb 2;11(2):e0147971. doi: 10.1371/journal.pone.0147971 (PMC4737494; doi:10.1371/journal.pone.0147971)
Supplement: S3 Fig — Non-metric multidimensional scaling (NMDS) ordination of phytochemical data obtained from seven populations of alfalfa (see Fig 1 in main text for population locations). Data consisted of peak intensity information for 49 compounds as characterized through HPLC analysis. The average value of a population on each NMDS dimension was calculated and plotted, lines emanating from this centroid extend to each datum (individual plant). Ordination demonstrated a high degree of phytochemical similarity between populations. (DOCX) [file pone.0147971.s004.docx]

S3 Figure. Non-metric multidimensional scaling (NMDS) ordination of phytochemical data obtained from seven populations of alfalfa (see Fig. 1 in main text for population locations). Data consisted of peak intensity information for 49 compounds as characterized through HPLC analysis. The average value of a population on each NMDS dimension was calculated and plotted, lines emanating from this centroid extend to each datum (individual plant). Ordination demonstrated a high degree of phytochemical similarity between populations.
