# Supplementary material for: The Many Dimensions of Diet Breadth: Phytochemical, Genetic, Behavioral, and Physiological Perspectives on the Interaction between a Native Herbivore and an Exotic Host
Source: PLoS One. 2016 Feb 2;11(2):e0147971. doi: 10.1371/journal.pone.0147971 (PMC4737494; doi:10.1371/journal.pone.0147971)
Supplement: S4 Fig — Phytochemical separation between alfalfa populations differing in L. melissa colonization. The top row depicts results of linear discriminant analysis. In the top left panel all 49 compounds characterized via HPLC were analyzed, while in the top right panel only those compounds occurring at all alfalfa populations were analyzed. Points are individual plants, triangles are used for alfalfa populations uncolonized by L. melissa, circles for colonized populations. The bottom left panel shows differences in compound concentration between colonized and uncolonized alfalfa populations. Differences in means shown reflect differences in mean peak intensity for a given compound. This frequency distribution only includes those compounds that occurred at all alfalfa populations analyzed. (DOCX) [file pone.0147971.s005.docx]

S4 Figure. Phytochemical separation between alfalfa populations differing in *L. melissa* colonization. The top row depicts results of linear discriminant analysis. In the top left panel all 49 compounds characterized via HPLC were analyzed, while in the top right panel only those compounds occurring at all alfalfa populations were analyzed. Points are individual plants, triangles are used for alfalfa populations uncolonized by *L. melissa,* circles for colonized populations. The bottom left panel shows differences in compound concentration between colonized and uncolonized alfalfa populations. Differences in means shown reflect differences in mean peak intensity for a given compound. This frequency distribution only includes those compounds that occurred at all alfalfa populations analyzed.
