# Supplementary material for: The Many Dimensions of Diet Breadth: Phytochemical, Genetic, Behavioral, and Physiological Perspectives on the Interaction between a Native Herbivore and an Exotic Host
Source: PLoS One. 2016 Feb 2;11(2):e0147971. doi: 10.1371/journal.pone.0147971 (PMC4737494; doi:10.1371/journal.pone.0147971)
Supplement: S5 Fig — Phytochemistry of surveyed alfalfa populations. The top row depicts results of linear discriminant analysis of 49 compounds characterized via HPLC (each combination of axes is a different combination of discriminant functions). Points are individual plants, triangles are used for alfalfa populations uncolonized by L. melissa, circles for colonized populations. Average values output by discriminant functions for a population are plotted by larval survival (row 2) and larval mass (row 3) to determine if the compounds that best differentiate alfalfa populations can predict the differences in larval performance, or colonization status, observed between those same populations. Discriminant functions proved poor predictors of larval performance. (DOCX) [file pone.0147971.s006.docx]

S5 Figure. Phytochemistry of surveyed alfalfa populations. The top row depicts results of linear discriminant analysis of 49 compounds characterized via HPLC (each combination of axes is a different combination of discriminant functions). Points are individual plants, triangles are used for alfalfa populations uncolonized by *L. melissa,* circles for colonized populations. Average values output by discriminant functions for a population are plotted by larval survival (row 2) and larval mass (row 3) to determine if the compounds that best differentiate alfalfa populations can predict the differences in larval performance, or colonization status, observed between those same populations. Discriminant functions proved poor predictors of larval performance.
