# Supplementary material for: The Many Dimensions of Diet Breadth: Phytochemical, Genetic, Behavioral, and Physiological Perspectives on the Interaction between a Native Herbivore and an Exotic Host
Source: PLoS One. 2016 Feb 2;11(2):e0147971. doi: 10.1371/journal.pone.0147971 (PMC4737494; doi:10.1371/journal.pone.0147971)
Supplement: S1 Table — Location and elevation of alfalfa (Medicago sativa) populations utilized for this study. (DOCX) [file pone.0147971.s007.docx]

| S1 Table. Location and elevation of alfalfa (*Medicago sativa*) populations utilized for this study. | | |
| --- | --- | --- |
|  |  |  |
| Population | Lat., Long. | Elevation (meters) |
| Beckwourth Pass (BWP) | 39.7797, -120.0734 | 1457 |
| Davis, CA (APLL) | 38.5820, -121.7498 | 15 |
| Fallon, NV (AFAL) | 39.4898, -118.5932 | 1197 |
| Gardnerville (GVL) | 38.8122, -119.7793 | 1554 |
| Silver Lake (SLA) | 39.6497, -119.9263 | 1512 |
| Star Creek Canyon (SCC) | 40.5496, -118.1143 | 1634 |
| Verdi (VUH) | 39.5099, -119.9950 | 1497 |
| White Fir (AWFS) | 39.5075, -119.8985 | 1422 |
