# Supplementary material for: The Many Dimensions of Diet Breadth: Phytochemical, Genetic, Behavioral, and Physiological Perspectives on the Interaction between a Native Herbivore and an Exotic Host
Source: PLoS One. 2016 Feb 2;11(2):e0147971. doi: 10.1371/journal.pone.0147971 (PMC4737494; doi:10.1371/journal.pone.0147971)
Supplement: S2 Table — Collection and sample size information for each oviposition assay performed. Each assay consisted of challenging a single female L. melissa butterfly with alfalfa from two populations and a negative control (Lotus nevadensis). All oviposition assays were conducted for 48 hours outdoors at ambient temperature. The two dates for Silver Lake were because inclement weather caused few eggs to be laid during the first assay of SLA females’ preference between AWFS and APPL. Consequently, two days later this assay was performed again using fresh females and data from the two assays were pooled. (DOCX) [file pone.0147971.s008.docx]

| S2 Table. Collection and sample size information for each oviposition assay performed. Each assay consisted of challenging a single female *L. melissa* butterfly with alfalfa from two populations and a negative control (*Lotus nevadensis*)*.* All oviposition assays were conducted for 48 hours outdoors at ambient temperature. The two dates for Silver Lake were because inclement weather caused few eggs to be laid during the first assay of SLA females’ preference between AWFS and APPL. Consequently, two days later this assay was performed again using fresh females and data from the two assays were pooled. | | | |
| --- | --- | --- | --- |
| Female source population | Sample size and collection date (numbers in parentheses are number of females that oviposited) | Total eggs | Mean eggs per ovipositing female (± SE) |
| AWFS vs. APLL vs. *Lotus nevadensis* | | | |
| Silver Lake, NV | 48 (14) (Aug 19 & 21, 2013) | 67 | 4.8 (1.3) |
| Verdi, NV | 46 (20) (Sept 5, 2013) | 135 | 6.8 (1.2) |
| AWFS vs. VUH vs. *Lotus nevadensis* | | | |
| Silver Lake, NV | 36 (13) (Sept 5, 2013) | 86 | 6.6 (1.8) |
| Verdi, NV | 47 (25) (Sept. 2, 2013) | 152 | 6.1 (1.0) |
