# Supplementary material for: The Many Dimensions of Diet Breadth: Phytochemical, Genetic, Behavioral, and Physiological Perspectives on the Interaction between a Native Herbivore and an Exotic Host
Source: PLoS One. 2016 Feb 2;11(2):e0147971. doi: 10.1371/journal.pone.0147971 (PMC4737494; doi:10.1371/journal.pone.0147971)
Supplement: S3 Table — Results from multiple mantel tests correlating a distance matrix of protein content with both a phytochemical distance matrix, and a genetic covariance matrix generated from alfalfa (Medicago sativa) individuals sourced from five populations (see main text for locations). Protein data was generated via a Bradford assay (absorbance/divided by mass). Phytochemistry data consisted of a matrix of peak intensity for 49 compounds (HPLC data again standardized by dry weight); and, genetic data consisted of a pairwise genetic covariance matrix (generated using 16,920 SNVs). All data were converted to distance matrices using a Euclidean distance measure, then analyzed with a multiple mantel test (1,000 permutations). Correlation coefficients using both Pearson’s product-moment correlation and Spearman’s rank correlation are given along with corresponding p values. (DOCX) [file pone.0147971.s009.docx]

| S3 Table. Results from multiple mantel tests correlating a distance matrix of protein content with both a phytochemical distance matrix, and a genetic covariance matrix generated from alfalfa (*Medicago sativa*) individuals sourced from five populations (see main text for locations). Protein data was generated via a Bradford assay (absorbance/divided by mass). Phytochemistry data consisted of a matrix of peak intensity for 49 compounds (HPLC data again standardized by dry weight); and, genetic data consisted of a pairwise genetic covariance matrix (generated using 16,920 SNVs). All data were converted to distance matrices using a Euclidean distance measure, then analyzed with a multiple mantel test (1,000 permutations). Correlation coefficients using both Pearson’s product-moment correlation and Spearman’s rank correlation are given along with corresponding p values. | | | | |
| --- | --- | --- | --- | --- |
| *Correlation with genetic distance* | | | | |
| Site | r (Pearson’s) | p | rho (Spearman’s) | p |
| AFAL | -0.24 | 0.70 | -0.3 | 0.80 |
| AWFS | -0.02 | 0.58 | -0.04 | 0.60 |
| VUH | 0.07 | 0.29 | -0.03 | 0.51 |
| GVL | 0.10 | 0.14 | 0.05 | 0.30 |
| SCC | 0.17 | 0.15 | 0.21 | 0.09 |
| *Correlation with phytochemical distance* | | | | |
| AFAL | -0.01 | 0.50 | 0.05 | 0.42 |
| AWFS | -0.14 | 0.85 | -0.06 | 0.67 |
| VUH | 0.04 | 0.32 | 0.15 | 0.18 |
| GVL | -0.11 | 0.77 | -0.01 | 0.52 |
| SCC | -0.19 | 0.95 | -0.16 | 0.91 |
