# Supplementary material for: The Many Dimensions of Diet Breadth: Phytochemical, Genetic, Behavioral, and Physiological Perspectives on the Interaction between a Native Herbivore and an Exotic Host
Source: PLoS One. 2016 Feb 2;11(2):e0147971. doi: 10.1371/journal.pone.0147971 (PMC4737494; doi:10.1371/journal.pone.0147971)
Supplement: S4 Table — Results from multiple Mantel test correlating phytochemical and genetic distance matrices generated from alfalfa (Medicago sativa) individuals sourced from five populations (see main text for locations). Phytochemistry data consisted of a matrix of peak intensity for 49 compounds (HPLC data standardized by dry weight); and, genetic data consisted of a pairwise genetic covariance matrix (generated using 16,920 SNVs). Both matrices were converted to distance matrices using a Euclidean distance measure, then analyzed with a multiple mantel test (1,000 permutations). Correlation coefficients using both Pearson’s product-moment correlation and Spearman’s rank correlation are given along with corresponding p values. (DOCX) [file pone.0147971.s010.docx]

| S4 Table. Results from multiple Mantel test correlating phytochemical and genetic distance matrices generated from alfalfa (*Medicago sativa*) individuals sourced from five populations (see main text for locations). Phytochemistry data consisted of a matrix of peak intensity for 49 compounds (HPLC data standardized by dry weight); and, genetic data consisted of a pairwise genetic covariance matrix (generated using 16,920 SNVs). Both matrices were converted to distance matrices using a Euclidean distance measure, then analyzed with a multiple mantel test (1,000 permutations). Correlation coefficients using both Pearson’s product-moment correlation and Spearman’s rank correlation are given along with corresponding p values. | | | | |
| --- | --- | --- | --- | --- |
| Site | r (Pearson’s) | p | rho (Spearman’s) | p |
| AFAL | -0.26 | 0.83 | -0.14 | 0.71 |
| AWFS | -0.01 | 0.55 | -0.02 | 0.53 |
| VUH | 0.13 | 0.21 | 0.18 | 0.14 |
| GVL | 0.27 | 0.04 | 0.21 | 0.06 |
| SCC | -0.14 | 0.81 | -0.20 | 0.90 |
