# Supplementary material for: The Many Dimensions of Diet Breadth: Phytochemical, Genetic, Behavioral, and Physiological Perspectives on the Interaction between a Native Herbivore and an Exotic Host
Source: PLoS One. 2016 Feb 2;11(2):e0147971. doi: 10.1371/journal.pone.0147971 (PMC4737494; doi:10.1371/journal.pone.0147971)
Supplement: S5 Table — Pairwise Fst values for alfalfa (Medicago sativa) populations examined. Populations prefixed by “A” were not colonized by L. melissa See main text for analytical and sequencing details. (DOCX) [file pone.0147971.s011.docx]

S5 Table. Pairwise Fst values for alfalfa (*Medicago sativa*) populations examined. Populations prefixed by “A” were not colonized by *L. melissa* See main text for analytical and sequencing details.

|  | AFAL | AWFS | GVL | SCC | VUH |
| --- | --- | --- | --- | --- | --- |
| AFAL | 0.000 | 0.012 | 0.012 | 0.010 | 0.019 |
| AWFS | 0.012 | 0.000 | 0.011 | 0.008 | 0.015 |
| GVL | 0.012 | 0.011 | 0.000 | 0.008 | 0.017 |
| SCC | 0.010 | 0.008 | 0.008 | 0.000 | 0.016 |
| VUH | 0.019 | 0.015 | 0.017 | 0.016 | 0.000 |
